# Molecular Mechanisms and Risk Factors for the Pathogenesis of Hydrocephalus

**DOI:** 10.3389/fgene.2021.777926

**Published:** 2022-01-03

**Authors:** Jingwen Li, Xinjie Zhang, Jian Guo, Chen Yu, Jun Yang

**Affiliations:** ^1^ Department of Neurosurgery, Xiang’an Hospital of Xiamen University, School of Medicine, Xiamen University, Xiamen, China; ^2^ Department of Neurosurgery, Tianjin Medical University General Hospital, Tianjin, China

**Keywords:** etiological study of hydrocephalus, molecular mechanism, risk factors, pathogenesis, CSF

## Abstract

Hydrocephalus is a neurological condition due to the aberrant circulation and/or obstruction of cerebrospinal fluid (CSF) flow with consequent enlargement of cerebral ventricular cavities. However, it is noticed that a lot of patients may still go through symptomatic progression despite standard shunting procedures, suggesting that hydrocephalus is far more complicated than a simple CSF circulative/obstructive disorder. Growing evidence indicates that genetic factors play a fundamental role in the pathogenesis of some hydrocephalus. Although the genetic research of hydrocephalus in humans is limited, many genetic loci of hydrocephalus have been defined in animal models. In general, the molecular abnormalities involved in the pathogenesis of hydrocephalus include brain development and ependymal cell dysfunction, apoptosis, inflammation, free radical generation, blood flow, and cerebral metabolism. Moreover, recent studies have indicated that the molecular abnormalities relevant to aberrant cerebral glymphatic drainage turn into an attractive subject in the CSF circulation disorder. Furthermore, the prevalent risk factors could facilitate the development of hydrocephalus. In this review, we elicited some possible fundamental molecular mechanisms and facilitating risk factors involved in the pathogenesis of hydrocephalus, and aimed to widen the diagnosis and therapeutic strategies for hydrocephalus management. Such knowledge could be used to improve patient care in different ways, such as early precise diagnosis and effective therapeutic regimens.

## Introduction

**FIGURE 1:**
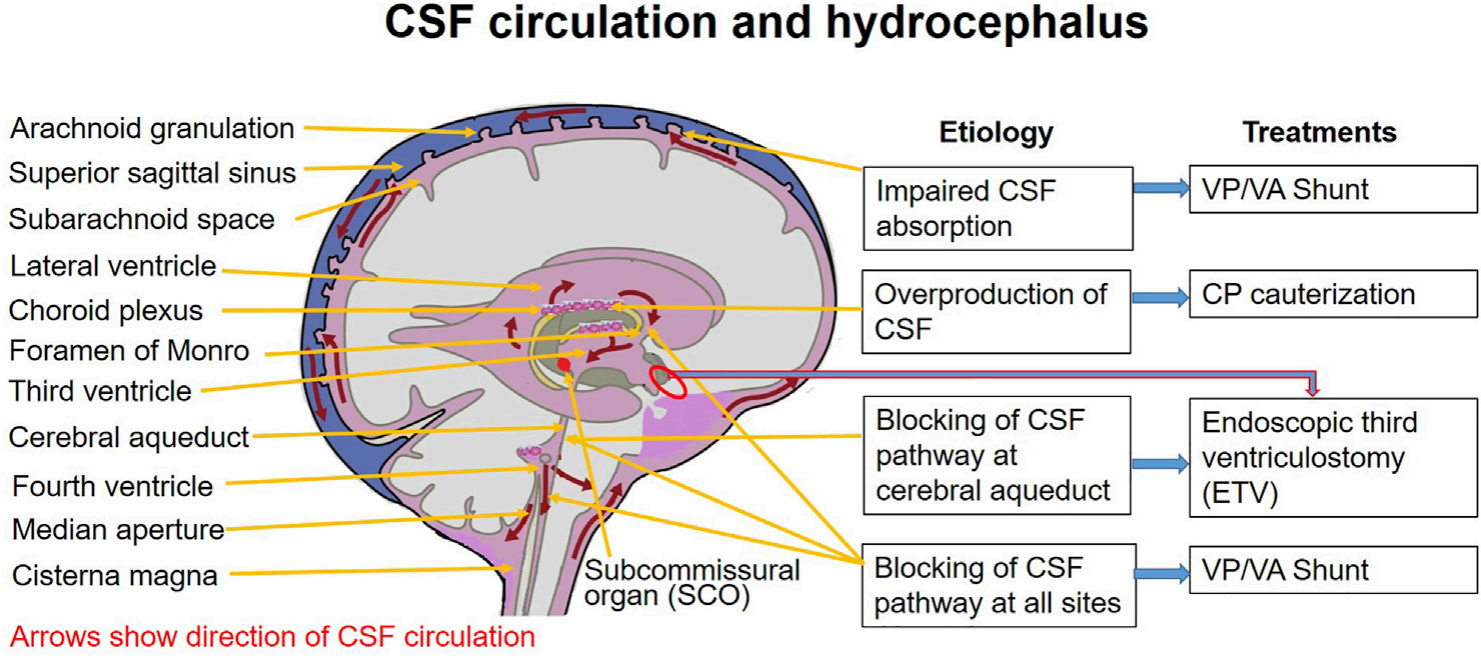
Hydrocephalus and therapeutic options. CP: choroid plexus; CSF: cerebrospinal fluid; EVT: endoscopic third ventriculostomy; VA: ventriculoatrial; VP: ventriculoperitoneal.

Hydrocephalus is defined as active distension of brain’s ventricular system, resulting from inadequate passage of cerebrospinal fluid from its point of production within the cerebral ventricles to its point of absorption into the systemic circulation (Rekate, 2008; Kousi and Katsanis, 2016). It is characterized by ventriculomegaly due to an imbalance between CSF production and its absorption. However, its precise causes remain largely unknown. Although the causes of obstructive hydrocephalus are not difficult to explore, the etiological factors of congenital and/or communicating hydrocephalus remain unclear. Moreover, up to 78% of hydrocephalus patients treated with functional shunting continue to suffer from cognitive function disorders, such as intellectual disability, memory loss, learning disabilities, and spasticity, suggesting that the etiology of hydrocephalus may be related to multiple factors (Villani et al., 1995; Silverberg et al., 2002; Zhang et al., 2020; Kundishora et al., 2021; Thavarajasingam et al., 2021). Accumulating evidence suggests that many molecular changes are involved in the pathogenesis of hydrocephalus, and about 40% of hydrocephalus may have a possible genetic etiology (Haverkamp et al., 1999; Zhang et al., 2006; Kundishora et al., 2021). Based on the genetic predisposition, acquired risk factors could expedite the progression of ventricular enlargement. Therefore, a clear understanding of the fundamental molecular and acquired risk factors of hydrocephalus is of critical importance for the study of its mechanism and adequate preventive measures. More importantly, a precise etiological diagnosis could facilitate optimal options for specific treatments of hydrocephalus; for example, CSF shunting vs. endoscopy vs. pharmacologic therapies (Figure 1; Table 1) (Koleva and De Jesus, 2021). Moreover, regarding a subset of hydrocephalus caused by impaired neurogenesis rather than active CSF accumulation, it is worth considering whether surgical CSF shunting is the therapeutic option for all hydrocephalus patients. In this review, we focused on the studies of the genetic and facilitating risk factors for hydrocephalus development, in animal as well as human studies, and examined the contributions of these molecular and risk factors in the pathogenesis and progression of hydrocephalus.

**TABLE 1:** Etiology and management options for hydrocephalus.

Hypersecretory	Obstructive	Communicating	iNPH	Congenital
Etiology of hydrocephalus				
Choroid plexus papilloma carcinoma	The obstructions are commonly caused by different tumors at foramina Monro, cerebral aqueduct, forth ventricle, foramen magnum, *etc*.	The most common causes are post-hemorrhagic, post-inflammatory, and post-TBI changes resulting in blocking of CSF absorption at the arachnoid granulations	A type of communicating hydrocephalus with increased incidence in older age with an unknown pathogenesis and no increase in ICP	Most caused by genetic-related congenital malformations and prenatal hemorrhage or infection
Management options				
Choroid plexus coagulation	Ventriculoperitoneal shunt, ventriculoatrial shunt, ventriculo-pleural shunt, lumboperitoneal shunt, and Torkildsen shunt. Endoscopic third ventriculostomy is used in aqueductal stenosis to avoid a permanent shunt			Multidisciplinary approach

## Animal Genetic Studies of Hydrocephalus

The genetic and histopathological similarities to human hydrocephalus made the animal model as a valuable tool to explore the pathogenesis of hydrocephalus. Most animal congenital hydrocephalus has been documented as genetic diseases, and many hydrocephalus-related loci have been identified (Table 2).

**TABLE 2:** Summary of genetic mechanism in the pathogenesis of hydrocephalus.

Disorder/etiopathogenesis	Species/strain	Genetic locus	Genetic trait/chromosome	Reference(s)
Hypersecretory: over production of CSF by choroid plexus	Human/mouse	E2f5; Tg737orpk	Autosomal recessive	Wagner et al. (2003); Banizs et al. (2005); Lindeman et al. (1998); Putoux et al. (2011)
Congenital/obstructive: defective ependymal cilia/flow/neural tube, and resultant closure of aqueduct	Human/mouse	Mdnah5, KIF7, VANGL1, 2, SMARCC1, TRIM71, PTCH1		Wagner et al. (2003); Banizs et al. (2005); Lindeman et al. (1998); Putoux et al. (2011); Fruey et al. (2018); Jin et al. (2020); Kibar et al. (2011); Mastromoro et al. (2021); Kundishora et al. (2021)
iNPH: dysfunction of the glymphatic pathway and sub-ischemia	Human/mouse	AQP4, Dp71		Eide and Hansson (2018)
Congenital/obstructive/communicating/iNPH: dysfunction of the glymphatic pathway due to aberrant motile cilium on ependymal cells	Human/mouse	Ccdc39, Celsr2, Celsr3, Cetn2, Dvls, FoxJ1, Hydin, Mdnah5, Pkd1, Tg737, Daple, Dnah14, Cfap43, Cwh43, FMN2, EML1, TMEM67, ZCCHC8		Kundishora et al. (2021); Yamada et al. (2021); Abdi et al. (2018); Wallmeier et al. (2019); Sotak and Gleeson (2012); Lechtreck et al. (2008); Takagishi et al. (2017); Morimoto et al. (2019); Yang et al. (2021); Law et al. (2014); Shaheen et al. (2017); McKnight et al. (2021)
Defective cilia orientation/aberrant CSF flow	Human	CCDC88C	Autosomal recessive	Marguet et al. (2021); Ohata et al. (2014)
Congenital: defective neural cell adhesion/stenosis of the aqueduct of Sylvius/agenesis of corpus callosum	Human	L1CAM	X-linked/Xq28	Adle-Biassette et al. (2013); Jouet et al. (1993); Okamoto et al. (2004); Willems et al. (1987); Bousquet et al. (2021); Wang et al. (2021); Etchegaray et al. (2020)
Aberrant vesicle trafficking	Human	AP1S2	X-linked	Kousi and Katsanis (2016); Marguet et al. (2021); Yang et al. (2019)
Disruption of the planar cell polarity pathway	Human	MPDZ	Autosomal recessive	Al-Jezawi et al. (2018); Sotak and Gleeson (2012)
Aqueductal stenosis without intellectual disability	Human	Unknown	Autosomal dominant/8q12.2-q21.2	Verhagen et al. (1998); Vincent et al. (1994)
SCO abnormalities/cerebral aqueduct closure/corpus callosum absence/free radical damage/abnormal cerebral hemisphere formation	Rat/HTX	Cck, Nfix, Xdh, Gsta1, Pax-6, Fkhr	Chr9, Chr10, Chr11, Chr17	Zhang et al. (2006); Miller et al. (2006); Oi et al. (1996); Somera and Jones (2004); Somera and Jones (2002)
Perturbation of growth factor signaling for cell function	Rat/HTX	TGFB, FGF-2, IGFBP-1, SOCS		Zhang et al. (2006); Li et al. (2005); Doublier et al. (2000); Ohmiya et al. (2001); Krebs et al. (2004)
Extracellular matrix disruption	Rat/HTX	TGFB1, TIMP-1		Zechel et al. (2002)
Aqueduct stenosis	Rat	LEW/Jms		Miller et al. (2006)
Symptomatic hydrocephalus of Dandy-Walker syndrome	Rat	6-AN		Oi et al. (1996)
Defective ependymal cell migration and proliferation/enhanced Notch signaling activity/aqueduct stenosis	Mouse	Rnd3		Lin et al. (2013)
Defective embryo development and ventricular size	Mouse	Vent8a, Vent4b, Vent7c	Chr4, Chr7, Chr8	Zhang et al. (2006); Zygourakis and Rosen (2003)
Defective cytoskeleton/ependymal cell malfunction	Human/Hy3 mouse	Bdnf, Mdnah5	Chr8	Zhang et al. (2006); Davy and Robinson (2003); Jimenez et al. (2001); Wagner et al. (2003); Ibanez-Tallon et al. (2004)
Defective mesenchymal cell/defective PC and SCO/collapse of the cerebral aqueduct	Mouse	Msx1, CYP2J2, RFX4_v3		Zhang et al. (2006); Blackshear et al. (2003)
Defective differentiation of arachnoid cells	Human/mouse	Mf1, FREAC3		Zhang et al. (2006); Kume et al. (1998)
Defective cellular membrane fusion/abnormal development of the neuronal cells	Hyh mouse	a-SNAP, aPKC, VAMP-7		Hong et al. (2004); Chae et al. (2004)
Intracranial hemorrhage and subcortical heterotopia	Mouse	Hhy	Chr12	Kuwamura et al. (2004)
Defective brain development and edematous periventricular white matter	Mouse	Otx2		Makiyama et al. (1997)

Abbreviations: 6-AN, 6-aminonicotinamide; AP1S2, adapter-related protein complex 1, sigma-2 subunit; aPKC, atypical protein kinase C; a-SNAP, soluble NSF attachment protein a; CCDC88C, coiled-coil domain-containing protein 88c; Cck, cholecystokinin; CSF, cerebrospinal fluid; Ch, congenital hydrocephalus; Chr, chromosome; E2f5, E2F transcription factor 5; FGF-2, fibroblast growth factor-2; Fkhr, fork-head transcription factor BF-1; Hhp, hemorrhagic hydrocephalus; Hyh, hydrocephalus and hop gait; Hy3, hydrocephalus-3; IGFBP-1, IGF binding protein-1; iNPH, idiopathic normal pressure hydrocephalus; L1CAM, L1 cell adhesion molecule; MPDZ, multiple PDZ domain protein; Mdnah5, axonemal heavy chain 5 gene; Mf1, Foxc1; Nfix, nuclear factor 1; PC, posterior commissure; Rnd3, Rho family guanosine triphosphatase 3; RFX4_v3, regulatory factor X4; SCO, subcommissural organ; SOCS, cytokine signaling protein; TGFB, transforming growth factor-beta; VAMP-7, vesicle-associated membrane protein-7; Wnt, wingless/integrated; Xdh, xanthine dehydrogenase.

### Rat Hydrocephalus Model

Three major congenital hydrocephalus models are Texas strain (HTX), LEW/Jms, and 6-aminonicotinamide (6-AN)-related hydrocephalus in rats. HTX rats proceed to cerebral aqueduct closure and consequently develop hydrocephalus. LEW/Jms rats demonstrate aqueduct stenosis and hydrocephalus in early prenatal life before pulmonary maturation. 6-AN–related hydrocephalus is similar to Dandy-Walker syndrome (Oi et al., 1996; Miller et al., 2006; Zhang et al., 2006).

The HTX rat is a well-studied congenital hydrocephalus model with ventricular enlargement in late gestation resulting from the cerebral aqueduct and subcommissural organ (SCO) abnormalities. SCO is a key structure for cerebral aqueduct patency and CSF flow. The reduced SCO glycoprotein level had been demonstrated to correlate to both aqueduct stenosis and enlarged lateral ventricle size in the HTX rats (Somera and Jones, 2002; Somera and Jones, 2004). Consistently, a human study of hydrocephalic fetuses also demonstrated a smaller SCO (Castaneyra-Perdomo et al., 1994). Following the development of cerebral aqueduct stenosis, the clinical feature manifests as early communicating to final obstructive hydrocephalus in fetuses (Oi et al., 1996; Miller et al., 2006).

The aqueduct closure in HTX rats may result from retrograde degeneration of apoptotic cells (Mori et al., 2002) and failure in cell proliferation (Draper et al., 2001; Mashayekhi et al., 2001) in the thalamus. The loci of correspondent genes have been indicated on chromosomes (Chr) 9 (peak markers D9Rat2), 10 (between markers D10Rat136 and D10Rat135), 11 (peak markers D11Arb2 and D11Rat46), and 17 (peak markers D17mit4 and D17Rat154), respectively (Zhang et al., 2006). Further genetic studies in the midbrain region of HTX rats showed that the related genes may include cholecystokinin (*Cck*), *Nfix*, Xanthine dehydrogenase (*Xdh*), *Gsta1*, *Pax-6*, and fork-head transcription factor BF-1 (*Fkhr*). Most of these genes have been investigated in clinical studies. For example, *Cck* levels decreased in hydrocephalus patients (Galard et al., 1997). *Cck* is known to be involved in learning and memory processes, and most hydrocephalic patients exhibit learning and memory deficits (Ding et al., 2001). *Nfix* is a member of the nuclear factor 1 (*Nf1*) family. *Nfix* deficiency causes corpus callosum absence and ventricular enlargement (Shu et al., 2003). Xanthine dehydrogenase catalyzes xanthine oxidation. The conversion of xanthine dehydrogenase to xanthine oxidase could lead to the concomitant generation of free radical products (Battelli et al., 1998). Gsta1 is an enzyme for free radical scavenging. The accumulation of free radicals is associated with the pathogenesis of hydrocephalus (Socci et al., 1999). Both *Pax-6* and *Fkhr* have been shown to be associated with hydrocephalus (Kume et al., 1998; Kume et al., 2000). Animals with *Pax-6* deficiency failed to develop the SCO, and *Fkhr* is known to be involved in the cerebral hemisphere formation (Hatini et al., 1999; Huh et al., 1999).

The hydrocephalic HXT rats expressed significantly higher transforming growth factor-beta (*Tgfb*) levels than their normal siblings, suggesting that the perturbation of growth factor signaling may also be involved in the pathogenesis of hydrocephalus (Li et al., 2005; Zhang et al., 2006). Together with other growth factors, such as fibroblast growth factor-2 (*FGF-2*) and IGF binding protein-1 (*IGFBP-1*), they are important cytokines and growth molecules in the brain, and play an essential role in cell proliferation, differentiation, survival, and motility. Hydrocephalus has been observed in animal models that overexpress these growth factors (Johanson et al., 1999; Doublier et al., 2000), possibly due to aberrant neuronal differentiation in the postnatal cerebral cortex. The proposed mechanisms may refer to the skeletal and predominant cranial changes which obstruct CSF flow and reduce CSF absorption into the systemic circulation due to increased venous pressure and overgrowth of aberrant brain compounds (Ohmiya et al., 2001; Li et al., 2005; Khonsari et al., 2012). In contrast, the loss of the suppressor of cytokine signaling (SOCS) protein function will lead to increased expression of cytokines and the development of hydrocephalus due to its inhibitory role (Krebs et al., 2004). Furthermore, extracellular matrix (ECM) disruption may also be involved in the pathogenesis of hydrocephalus. In the *Tgfb1* overexpression model, the changes of matrix metalloproteinase-9 (Mmp-9) and its inhibitor Timp-1 were found to be important factors for developing hydrocephalus due to ECM environment alterations (Zechel et al., 2002).

Another congenital hydrocephalus strain, LEW/Jms rats, manifests as a similar strain with the HTX rats. The inheritance of hydrocephalus in these rats is possibly autosomal recessive or semidominant, but none of the loci has been identified (Jones et al., 2003).

### Mouse Hydrocephalus Model

Hydrocephalus could result from aqueductal stenosis induced through genetic deletion of Rho family guanosine triphosphatase 3 (*Rnd3*) and regulating the Notch signaling activity (Lin et al., 2013). *Rnd3* regulates cell migration and proliferation, and cell actin cytoskeleton dynamics. Deletion of the *Rnd3* gene results in severe obstructive hydrocephalus with enlargements of the lateral and third ventricles, but normal fourth ventricle size, indicating the blocking of the cerebral aqueduct. The molecular mechanism was reported to be *Rnd3* deficiency and consequent enhancement of Notch signaling activity, resulting in the overgrowth of aqueduct ependymal cells and obstructive hydrocephalus. Accordingly, this type of hydrocephalus could be resolved by the inhibition of Notch activity.

Quantitative trait locus (QTL) genetic mapping had been performed in mouse models, and three QTL loci were identified on Chr 8, Chr 4, and Chr 7, and labeled as *Vent8a*, *Vent4b*, and *Vent7c*, respectively. They are associated with congenital hydrocephalus by affecting ventricular size in the developing embryo (Zygourakis and Rosen, 2003; Zhang et al., 2006).

Although the autosomal recessive congenital hydrocephalus-1 (*hy1*) and hydrocephalus-2 (*hy2*) mice have been reported, the genetic loci have not been identified (Zhang et al., 2006). In contrast, the autosomal recessive mutation hydrocephalus-3 (*hy3*) mouse has been investigated comprehensively. A *hy3* mouse manifests severe communicating hydrocephalus in the perinatal stage. Another transgenic mouse line OVE459 is caused by a *Bdnf* transgene–induced insertional mutation on a single locus on chromosome 8, and the insertion locus is overlapped with that of *hy3*. The transgene insertion resulted in a rearrangement of the *hy3* gene exons in OVE459 mice (Zhang et al., 2006). Hy3 protein (Hydin) is homologous to Caldesmon, an actin-binding protein, suggesting that Hydin interacts with the cytoskeleton (Davy and Robinson, 2003). In the ventricular system of *hy3* mice, Hydin is confined to the ciliated ependymal cell layer. Studies demonstrate that ependymal cell malfunction may contribute to hydrocephalus (Jimenez et al., 2001; Wagner et al., 2003). The protein Dynein, coded by axonemal heavy chain 5 gene (*Mdnah5*), is also specifically expressed in ependymal cells and is essential for the structure and function of ependymal cilia. In *Mdnah5*-mutant mice, the ependymal flow disturbance and consequent aqueduct closure finally result in the development of hydrocephalus. In hydrocephalus patients, the correlation of ciliary defect and aqueductal stenosis also proves the relevance of this pathogenesis in humans (Ibanez-Tallon et al., 2004).

Hydrocephalus may also result from the abnormality of mesenchymal cells. In mice, the *Msx1* gene is expressed along the dorsal midline and involved in epithelial–mesenchymal interactions in organogenesis. The mouse with homozygous *Msx1* mutants showed malformation or absence of posterior commissure (PC) and SCO, and subsequently developed the collapse of the cerebral aqueduct and hydrocephalus. Failure of SCO development can also be caused by inserting the *Cyp2j2* transgene to interfere with the regulatory factor X4 (*Rfx4*) (Blackshear et al., 2003). Disruption of *Rfx4* can reduce *Msx2* expression and subsequently prevent the formation of SCO, and finally lead to hydrocephalus development.

Spontaneous congenital hydrocephalus (ch) can also be caused by mutation of gene *Mf1* (*Foxc1*) (Kume et al., 1998; Zhang et al., 2006). *Mf1*-encoded protein is expressed in many embryonic tissues, including mesenchyme, endothelial cells, and meninges. Mice with *Mf1* mutation were found to show multiple developmental disorders, including abnormal differentiation of arachnoid cells in meninges. In addition, patients with deletion of *FREAC3*, a homolog of *Mf1*, were also found to develop hydrocephalus (Kume et al., 1998).

A mouse with autosomal recessive hydrocephalus and hop gait (*Hyh*) manifests ventricle enlargement and hop gait at birth. The *Hyh* gene was identified as soluble NSF-attachment protein α (α-Snap) (Hong et al., 2004). α-Snap plays an important role in cellular membrane fusion (Chae et al., 2004). The membrane fusion is essential for inter- and intracellular molecular transportation and communication (Hong et al., 2004). In the *Hyh* mouse, abnormal localization of many apical proteins was identified; these proteins including SNAP receptor (Snare), beta-catenin, E-cadherin, vesicle-associated membrane protein-7 (Vamp-7), and atypical protein kinase C (aPkc) (Chae et al., 2004). The *Hyh* mouse demonstrated a small cerebral cortex and died postnatally from deteriorative hydrocephalus. The small cortex exhibited an abnormal development of the neuronal cells.

Another autosomal recessive mouse model is hemorrhagic hydrocephalus (*Hhy*). The *Hhy* locus has been localized on Chr 12, and the mouse is characterized by intracranial hemorrhage, hydrocephalus, and subcortical heterotopia (Kuwamura et al., 2004). The mouse manifested as communicating hydrocephalus with patent aqueduct and no histological abnormalities in choroid plexus and subarachnoid space. Following the development of hydrocephalus, numerous hemorrhages throughout the brain parenchyma and meninges could be observed.

Autosomal dominant hydrocephalus can be caused by *Otx2* mutation. The mouse was characterized by a lateral ventricle dilation and a ballooned cerebrum. The histological examination showed edematous changes in periventricular white matter, suggesting that the function of *Otx2* may have been a brain developmental organizer (Makiyama et al., 1997).

## Human Genetic Studies of Hydrocephalus

### Congenital Hydrocephalus Studies

Congenital hydrocephalus could be related to abnormal brain development and cellular dysfunction, suggesting genetic involvement in the pathogenesis of hydrocephalus (Stoll et al., 1992). It is estimated that up to 40% of cases of hydrocephalus refer to a possible genetic etiology (Haverkamp et al., 1999), and over 100 genes have been described to be mutated in syndromic hydrocephalus cases (Kousi and Katsanis, 2016). Thereinto, X-linked hydrocephalus comprises about 5–15% of genetic-related congenital hydrocephalus (Haverkamp et al., 1999; Zhang et al., 2006; Adle-Biassette et al., 2013), and bona fide mutations have been described in four genes, that is, L1 cell adhesion molecule (*L1CAM*), sigma 2 subunit of the adapter protein 1 complex (*AP1S2*), multiple PDZ domain proteins (*MPDZ*), and coiled-coil domain-containing protein 88c (*CCDC88C*) (Tarpey et al., 2006; Al-Dosari et al., 2013; Shaheen et al., 2017; Yang et al., 2019; Etchegaray et al., 2020; Marguet et al., 2021; Wang et al., 2021). Since the phenotypes are heterogenic, there may be more genetic loci in congenital hydrocephalus. Although the loci or genes for congenital hydrocephalus have not yet been identified fully, the mutations of the respondent genes may be expressed as X-linked and/or autosomal dominant in fashion.

The genes responsible for X-linked human congenital hydrocephalus are *L1CAM* (L1 protein), located at Xq28 (Jouet et al., 1993; Adle-Biassette et al., 2013), and *AP1S2* (Shaheen et al., 2017; Marguet et al., 2021). The exact mechanisms of the gene mutation and consequent L1 protein dysfunction are still not clear. However, a possible correlation between L1 protein dysfunction and the pathogenesis of hydrocephalus is the disruption of neural cell membrane proteins that play an important role during brain development. The L1 protein is expressed in neurons and Schwann cells and is a member of the immunoglobulin family for neural cell adhesion (Okamoto et al., 2004). As a neuronal adhesion molecule, *L1CAM* mediates functions of cell–cell adhesion; growth-cone morphology; the guidance of neurite outgrowth, myelination, axon bundling, and pathfinding; long-term potentiation, neuronal cell survival and migration, and synaptogenesis (Adle-Biassette et al., 2013). Mutations in *L1CAM* cause hydrocephalus with stenosis of the aqueduct of Sylvius and agenesis of the corpus callosum, hypoplasia of corticospinal tracts and the anterior cerebellar vermis, fusion of the thalami, and spastic paraplegia (Willems et al., 1987; Bousquet et al., 2021). The precise mechanism of *L1CAM* defect–related ventricular dilation remains unclear, and the hypotheses are possibly due to *L1CAM*-mediated decrease in white matter elasticity, increased CSF pressure and ventricular vulnerability, abnormal development of the midline structure, and narrowing of the CSF pathway (Kousi and Katsanis, 2016). *AP1S2* encodes the sigma 2 subunit of the AP1 Adaptin protein for regulating lysosomal protein sorting, and the mutation of *AP1S2* was reported to relate to X-linked congenital hydrocephalus through aberrant vesicle trafficking (Kousi and Katsanis, 2016; Shaheen et al., 2017; Marguet et al., 2021).

*CCDC88C* encodes DAPLE protein and acts as a negative regulator of the non-canonical wingless/integrated (WNT) signaling pathway; its mutation results in hydrocephalus through defective cilia orientation and aberrant CSF flow (Ohata et al., 2014; Marguet et al., 2021). *MPDZ* was reported to cause autosomal recessive non-syndromic communicating hydrocephalus through the disruption of the planar cell polarity pathway (Al-Jezawi et al., 2018).

Autosomal dominant congenital hydrocephalus was reported in one kindred. In contrast to X-linked or recessive congenital hydrocephalus with stenosis of the aqueduct of Sylvius, these patients were also associated with aqueductal stenosis but not related to intellectual disability and pyramidal tract dysfunction (Verhagen et al., 1998). Another hydrocephalus kindred with an identified microdeletion of 8q12.2-q21.2 was also transmitted in an autosomal dominant fashion (Vincent et al., 1994). In dystroglycanopathies, hydrocephalus is driven by aqueduct obstruction due to aberrational integrity of basement membranes in the brain, caused by mutant genes encoding for glycosyltransferases, that is, protein O-mannosyltransferase 1 (*POMT1*), Fukutin-related protein (*FKRP*), acetylglucosaminyltransferase-like protein (*LARGE*), and protein kinase B (*AKT*) (Langenbach and Rando, 2002; Akasaka-Manya et al., 2004; Satz et al., 2008). For syndromic hydrocephalus, the pathogenic mechanisms may relate to neural tube defects with resultant ependymal cilium dysfunction and aqueduct stenosis, and the mutant genes may include *vang-like1*, *2* (*VANGL1, 2*), *SMARCC1*, *TRIM71*, and *PTCH1* (Kibar et al., 2011; Furey et al., 2018; Jin et al., 2020; Kundishora et al., 2021; Mastromoro et al., 2021). Both *PIK3CA* and *PTEN* genes connected with the PI3K–AKT–mTOR pathway are correlated with the pathogenesis of hydrocephalus through the mechanisms of cerebellar overgrowth and obstruction of CSF flow (Jin et al., 2020; Mastromoro et al., 2021).

### iNPH Studies

In contrast to congenital hydrocephalus, acquired or adult-onset idiopathic normal pressure hydrocephalus (iNPH) is characterized by gait disturbance, urinary incontinence, and cognitive impairment. Adult-onset hydrocephalus may develop either due to decompensation of congenital hydrocephalus or sequel to an acquired CSF circulation disturbance. The acquired CSF circulation disorder may be due to late-onset aqueductal stenosis or disruption of CSF absorption (Chahlavi et al., 2001; Edwards et al., 2004). Although an X-linked adult-onset iNPH (Katsuragi et al., 2000) and an autosomal dominant fashion of familial NPH (Portenoy et al., 1984) have been reported, the related genetic studies have not been carried out yet.

### Glymphatic System and Hydrocephalus

**FIGURE 2:**
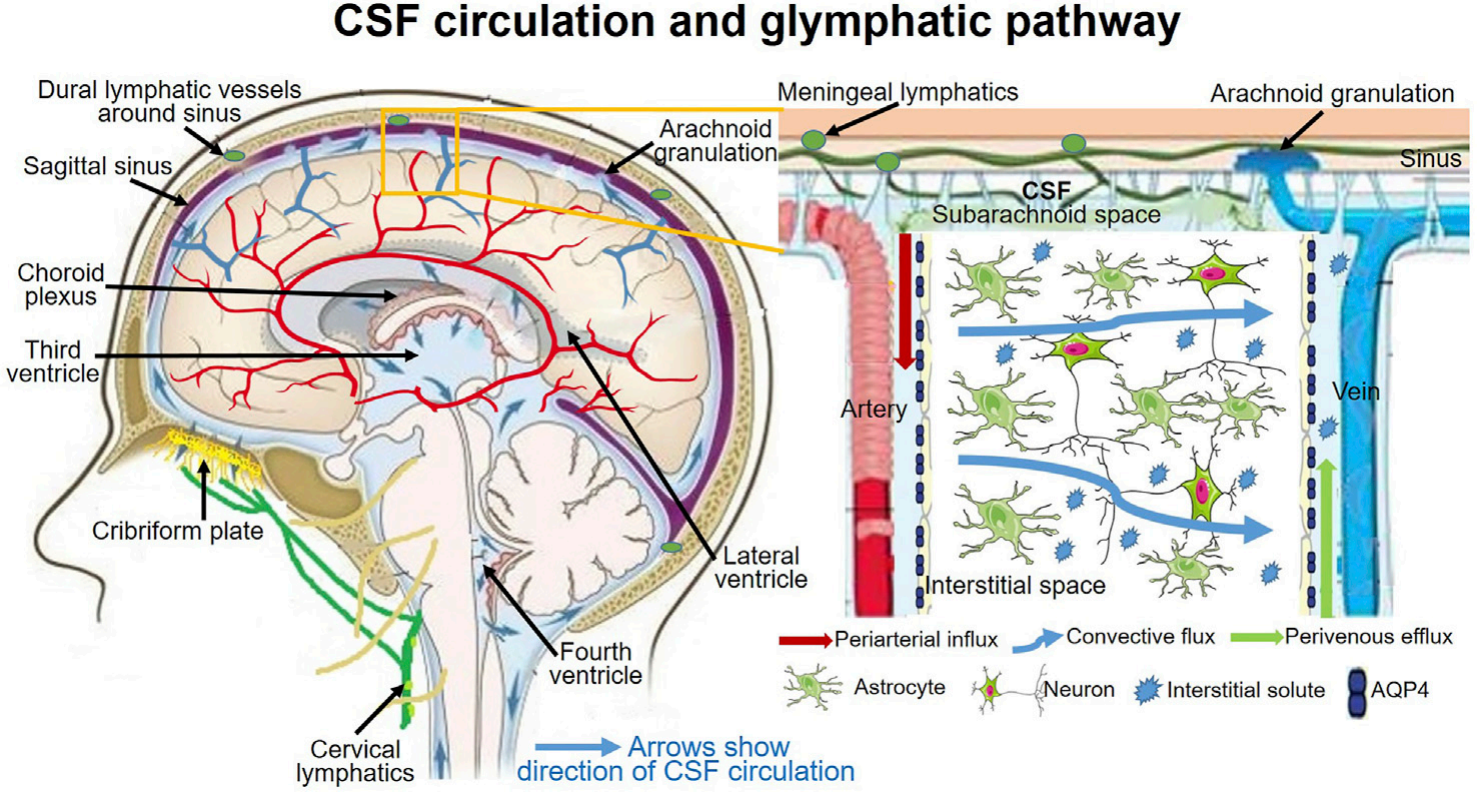
CSF circulation and glymphagitc pathway.

Challenging to traditional CSF physiology, increasing investigations raise the new concept of CSF dynamics that, rather than choroid plexus, CSF production also comes from the interstitial fluid efflux, and the major CSF outflow pathway may go through the lymphatic drainages rather than dural venous sinuses through arachnoid villi (Louveau et al., 2016; Da Mesquita et al., 2018; Louveau et al., 2018; Mestre et al., 2018; Ringstad and Eide, 2020; Yamada et al., 2021). The CSF outflow through lymphatic drainages mainly goes through the subarachnoid spaces to the meningeal lymphatics and cervical lymphatics *via* the cribriform plate and nasal lymphatics (Proulx, 2021). This new CSF dynamic concept has been termed as the glymphatic system (Figure 2).

The glial-lymphatic or glymphatic system is a recently identified fluid clearance system with a highly-organized transport pathway. This pathway subserves the flow of CSF from the subarachnoid space into the brain through perivascular spaces of the large leptomeningeal and penetrating arteries (Iliff et al., 2012; Ma et al., 2017), also known as Virchow-Robins spaces (Iliff et al., 2012; Pizzo et al., 2018); and thence into the brain interstitium. In the interstitium, the fluid disperses *via* a polarized net fluid movement toward the venous perivascular and perineuronal spaces (Iliff et al., 2012). Meanwhile, the CSF flows across the glial basement membrane and astroglial end-feet fringing the brain parenchyma (Iliff et al., 2012). In the astrocytic end-feet, the water channel aquaporin-4 (AQP4) is highly expressed and facilitates the flow of CSF into the brain parenchyma, where it mixes with the interstitial fluid (ISF) (Iliff et al., 2012; Kress et al., 2014). The pathway finally directs the flow toward the venous perivascular space, perineuronal sheaths of cranial and spinal nerves, meningeal lymphatic vessels, and arachnoid granulations, ultimately clearing solutes from the neuropil into meningeal and cervical lymphatic drainage vessels (Figure 2) (Louveau et al., 2015; Absinta et al., 2017; Ma et al., 2017).

In rodents, the glymphatic pathway is primarily active during sleep, when the clearance of harmful metabolites such as amyloid β (Aβ) increases two-fold relative to the waking state (Rasmussen et al., 2018; Hablitz and Nedergaard, 2021). However, this action decreases in most iNPH (Ringstad et al., 2017; Eide and Ringstad, 2019) due to the high incidence of sleep disorders, such as obstructive sleep apnea, in iNPH patients (Roman et al., 2018). The glymphatic dysfunction in iNPH patients was supposed to be caused by inefficient arterial pulsation due to excessive breathing during obstructive sleep apnea (Roman et al., 2019), resulting in decreased brain fluid and cerebral metabolite clearance through paravascular exchange of CSF and ISF. This paravascular CSF-ISF exchange increases by 60% during sleep compared with being awake (Xie et al., 2013). Moreover, increased awakenings during obstructive sleep apnea disturb sleep-associated circulation of interstitial CSF into the glymphatic flux, further contributing to hydrocephalus. In addition, glymphatic dysfunction resulted from the blockage of the airway during obstructive sleep apnea was reported to be associated with the aggregation of Aβ and other toxic solutes, which is closely linked with cognitive impairment as manifested in iNPH and other dementia diseases (Ju et al., 2019). Aβ is degraded and cleared *via* multiple pathways (Tarasoff-Conway et al., 2015), and the glymphatic pathway is a significant factor in the net clearance of Aβ (Xu et al., 2015). Impaired glymphatic function in *AQP4* knockout mice resulted in a 55% reduction in the parenchymal clearance of radio-labeled Aβ (Iliff et al., 2012) due to reduced efflux of ISF to the CSF (Xu et al., 2015). Furthermore, some iNPH patients responding to CSF diversion were presented with persistent disorders of cognitive function and abnormal pulsatile ICP (Villani et al., 1995; Silverberg et al., 2002; Zhang et al., 2020; Kundishora et al., 2021; Thavarajasingam et al., 2021), indicating impaired intracranial compliance and underlying pathological changes. A main histopathological finding was astrogliosis and reduction of AQP4 and of Dp71 in astrocytic perivascular end-feet. The altered AQP4 and Dp71 complex possibly contributed to the sub-ischemia and related pathological changes prevalent in the brain tissue of iNPH (Eide and Hansson, 2018). Therefore, the dysfunction of the glymphatic system may play an important role in the pathogenesis of iNPH patients.

As an essential constituent of the glymphatic system, ependymal cilia line the ventricles and interventricular connections. One of the critical functions of ependymal cilia is to effectively drive directional CSF ependymal flow through a regular synchronous beating in the glymphatic pathway (Ibanez-Tallon et al., 2004). In addition, during brain development, the patency of the aqueduct also requires an ependymal flow to maintain, and any abnormality of this flow can lead to aqueduct stenosis and hydrocephalus subsequently. Moreover, cilia also play a role in the regulation of CSF production. Therefore, aberrant molecular changes of ependymal cilia have closely related to the pathogenesis of hydrocephalus through the glymphatic mechanism. For example, mutations of *Mdnah5* and *Kif7* will lead to lack of ependymal flow and closure of aqueduct, and dysfunctions of Tg737orpk and E2F transcription factor 5 (E2f5) will result in CSF overproduction through the increased secretory activity of the choroid plexus; both abnormalities will result in hydrocephalus (Lindeman et al., 1998; Ibanez-Tallon et al., 2004; Banizs et al., 2005; Putoux et al., 2011).

The tight interplay between primary cilia and centrosomes makes it difficult to allocate independent composition and role to either organelle in the processes of neocortical development (Wilsch-Brauninger and Huttner, 2021). The centrosome plays a major role in the primary microtubule organization. Its dysfunction has a major impact on brain size and functionality, including ciliogenesis, suggesting a possible involvement in the glymphatic system (Marthiens and Basto, 2020; Yang et al., 2021b; Narita and Takeda, 2021). The centrosome is a complex organelle composed of two perpendicular cylindrical microtubule-based structures termed as the proximal and distal ends or centrioles. The proximal end of the centrosome is critical for pericentriolar material recruitment and microtubule-organizing activity, whereas the distal end is essential for appendage formation, membrane docking, and primary ciliogenesis (Paintrand et al., 1992). Based on its property of specialized structures and a large collection of proteins and other molecules, centrosomes exhibit a highly orchestrated biogenesis cycle. Any error in their regulation may lead to structurally and functionally compromised centrosomes and subsequent disorders. For example, the formation of the functional primary cilia depends on the intraflagellar transport (IFT) system (Pedersen et al., 2008). *Ift88* is a crucial member of the IFT-B complex, involved in anterograde transport. Kinesin family member 3a (*Kif3a*) is a subunit of the heterotrimeric kinesin complex and is responsible for anterograde transport too. *Arl13b* encodes a ciliary localized small GTPase of the ARF/ARL family, being essential for ciliary axoneme structure and signaling (Caspary et al., 2007). The genetic ablations of *Kif3a*, *Ift88*, and *Arl13b* at early stages of cortical development lead to a generally dysmorphic telencephalon including reduced cortical neurogenesis, lack of olfactory bulbs, and ventriculomegaly (Willaredt et al., 2008; Higginbotham et al., 2013; Insolera et al., 2014; Snedeker et al., 2017; Yang et al., 2021b). Notably, the genetic ablations of *Ift88* or *Kif3a* in the neural crest and midbrain also lead to a significant cortical malformation and ventriculomegaly (Snedeker et al., 2017), suggesting the requirement of primary cilia in non-forebrain tissue to regulate cortical development and ventricle size. In addition, a previous study showed that deletion of *Kif3a* using *Nestin-Cre* led to cortical disorganization and robust ventriculomegaly (Wilson et al., 2012). Remarkably, the *Nes-Cre8* transgene is widely expressed in both forebrain and surrounding non-forebrain tissues, indicating a contribution of cortical and non-cortical ciliary defects in this mutant (Petersen et al., 2002). Moreover, mutation of Polo-like kinase 4 (*Plk4*) gene and resultant centrosome dysfunction and cilium defect have been identified as causes in the pathophysiology of autosomal recessive developmental disorders, Seckel syndrome (SCKL) (Martin et al., 2014). SCKL patients show short stature and brain structural anomalies (Griffith et al., 2008; Martin et al., 2014; Gambarotto et al., 2019). These findings suggest that centrosome membrane anchorage, in the process of ciliogenesis, plays a vital role in regulating cortical development and ventricle size.

Based on the new CSF dynamic concept, the pathogenic mechanisms of iNPH or communicating hydrocephalus that correlate with the alterations in motile cilia and cerebrospinal fluid dynamics have been extensively investigated, and the related mutant genes have also been reported (Table 2). The mutant genes related to motile cilium dysfunction on ependymal cells, and consequent hydrocephalus development may include *Ccdc39*, *Celsr2*, *Celsr3*, *Cetn2*, *Dvl*, *Foxj1*, *Hydin*, *Mdnah5*, *Pkd1*, *Tg737*, *Daple*, *Dnah14*, *Cfap43*, *Cwh43*, *Eml1*, *Fmn2*, *Tmem67*, and *Zcchc8* (Law et al., 2014; Shaheen et al., 2017; Kundishora et al., 2021; McKnight et al., 2021; Yamada et al., 2021). For example, *Foxj1* mutation results in ciliopathy and hydrocephalus due to dysfunction of motile cilia (Abdi et al., 2018; Wallmeier et al., 2019). *Celsr2* is a planar cell polarity gene, and mutation results in hydrocephalus through ependymal cilia dysfunction (Sotak and Gleeson, 2012). Mutation of gene *Hydin*, which encodes a key protein within motile cilia in the ventricles, may also cause hydrocephalus (Lechtreck et al., 2008). *Daple* regulates the direction of CSF flow through motile cilium beating in the ependymal cells, and mutation of *Daple* results in communication hydrocephalus (Takagishi et al., 2017). Mutation of *Cfap43* causes a morphologic abnormality of ependymal cilia and consequent hydrocephalus (Morimoto et al., 2019). *Cwh43* modifies the glycosylphosphatidylinositol-anchored proteins on the ependymal cells, and the mutant *Cwh43* is related to iNPH in both humans and mice. The clinical features manifest as late-onset communicating hydrocephalus with symptoms of gait and balance dysfunction (Yang et al., 2021a).

The clinical manifestation and progression, as well as experimental investigations, indicate that hydrocephalus is a complex disease with polygenic involvement, rather than a simple CSF accumulation disorder. Although the current studies have revealed that some genetic mutations are involved in the pathogenesis of hydrocephalus, how these mutations are associated with the disorder of CSF circulation and their pathogenic roles in the pathological progression of hydrocephalus still remain largely unknown. Previous studies indicated that a lot of genetic mutations were relevant to the disorders of ciliary and/or centrosome, resulting in the dysfunction of the glymphatic system. However, how these mutations and their interactions contribute to the pathogenesis of hydrocephalus needs to be further elucidated. Moreover, there is still a lack of basic knowledge on the mechanisms underlying the cognitive functional impairment of hydrocephalus. Therefore, further extensive studies should be conducted to explore the underlying molecular mechanisms of identified and/or unidentified genes in the pathophysiology of hydrocephalus. Based on our knowledge, we propose that the genetic mutations relevant to ciliary and centrosomal proteins and the interaction between glymphatic system and ciliary/centrosomal structures/functions may be a critical molecular mechanism in the pathophysiology of hydrocephalus. In addition, based on these fundamental molecular mechanisms, it is noteworthy that environmental and other acquired risks or etiological factors are also involved in the facilitation of ventricular enlargement.

### The Role of Environment and Acquired Risk Factors in the Pathogenesis of Hydrocephalus

With regard to the environment and acquired elements, alcohol consumption, diabetes, hypertension, inflammation, aging, and sleep apnea are remarkable risk factors for iNPH development (Sakata-Haga et al., 2004; Kahle et al., 2016; Omran et al., 2017; Holth et al., 2019; Ghaffari-Rafi et al., 2020; Zhang et al., 2020; Yamada et al., 2021). Alcohol consumption demonstrated a correlation with iNPH development possibly due to ethanol-induced decrease in beating frequency of motile cilia and consequent dysfunction on the ependymal cells of ventricles (Omran et al., 2017; Ghaffari-Rafi et al., 2020). Although iNPH patients present with increased morbidity of diabetes, the causal relationship has not been concluded (Hudson et al., 2019; Rasanen et al., 2020). The speculative pathogenesis may be attributed to high concentration of sugar and increased fluid viscosity in CSF. The studies have indicated that increased fluid viscosity increases the shear stress on the ventricular wall and accelerates the dilatation of the vasculature (Roux et al., 2020). Hypertension and aging were also reported to be potential risk factors for ventriculomegaly. The underlining mechanism was considered to be related to the decline in the glymphatic function (Mestre et al., 2018; Ringstad and Eide, 2020). As previously discussed, obstructive sleep apnea was frequently associated with iNPH (Roman et al., 2018), and up to 90% of iNPH patients suffered from obstructive sleep apnea (Roman et al., 2019). In addition to the previously discussed pathogenic mechanism, obstructive sleep apnea reduces oxygen intake and decreases venous return to the heart, resulting in further retrograde intracranial venous hypertension and ventricular enlargement. It is worth noting that sleep and glymphatic system have become critical elements in the pathogenesis of iNPH, and they may be possibly manipulated through the changes of cardiac-induced arterial pulsation and intracranial pressure pulsatility with the cardiac-gated device used in our previous studies (Iliff et al., 2013; Luciano et al., 2017).

## Conclusion and Future Perspectives

In summary, it is essential to recognize that the current studies of genetic foundation and predisposing risk factors are promising approaches toward addressing the usual concern about whether an observed phenomenon is a consequence or a cause of hydrocephalus. Although the genetic study of hydrocephalus in humans is limited, many genetic loci of hydrocephalus have been identified in animal models. This approach offers researchers significant insights into the molecular etiology of impaired brains during the pathogenesis of hydrocephalus. In addition, genetic conditions affecting motile cilia lay the foundation, and acquired risk factors facilitate the development of hydrocephalus. Thereinto, the glymphatic system plays a key role in the activities of brain’s physiological metabolism and pathological process of hydrocephalus. Moreover, dysfunction of the glymphatic system and sleep disorders are emerging as popular topics in the studies of pathogenesis and possible therapeutic management of hydrocephalus, especially for iNPH. Therefore, these types of studies will facilitate a greater understanding of the molecular mechanism in hydrocephalus and provide valuable insights into the pathogenesis of iNPH and other neurological diseases. Possible new mechanisms other than altered CSF circulation and resorption, if uncovered *via* genetic research, may also help to explain why patients with hydrocephalus may still experience symptomatic progression despite functioning shunts. Ultimately, such knowledge will be useful in the improvement of patient care in different ways and also guide the optimal treatment decisions for patients as early as possible.
